# Proteomic analysis of extracellular vesicles from a *Plasmodium falciparum* Kenyan clinical isolate defines a core parasite secretome

**DOI:** 10.12688/wellcomeopenres.11910.2

**Published:** 2017-11-22

**Authors:** Abdirahman Abdi, Lu Yu, David Goulding, Martin K. Rono, Philip Bejon, Jyoti Choudhary, Julian Rayner

**Affiliations:** 1 Pwani University Bioscience Research Centre, Pwani University, Kilifi, Kenya; 2 KEMRI-Wellcome Trust Research Programme, Kilifi, Kenya; 3 Wellcome Trust Sanger Institute, Wellcome Genome Campus, Hinxton, Cambridge, UK

**Keywords:** Malaria, Plasmodium falciparum, extracellular vesicles, exosomes, proteomics

## Abstract

**Background**: Many pathogens secrete effector molecules to subvert host immune responses, to acquire nutrients, and/or to prepare host cells for invasion. One of the ways that effector molecules are secreted is through extracellular vesicles (EVs) such as exosomes. Recently, the malaria parasite *P. falciparum* has been shown to produce EVs that can mediate transfer of genetic material between parasites and induce sexual commitment. Characterizing the content of these vesicles may improve our understanding of *P. falciparum* pathogenesis and virulence.

**Methods**: Previous studies of *P. falciparum *EVs have been limited to long-term adapted laboratory isolates. In this study, we isolated EVs from a Kenyan *P. falciparum* clinical isolate that had been adapted to *in vitro* culture for a relatively shorter period, and characterized their protein content by mass spectrometry (data are available via ProteomeXchange, with identifier PXD006925).

**Results**: We show that *P. falciparum* extracellular vesicles ( *Pf*EVs) are enriched in proteins found within the exomembrane compartments of infected erythrocytes such as Maurer’s clefts (MCs), as well as the secretory endomembrane compartments in the apical end of the merozoites, suggesting that *Pf*EVs may play a role in parasite-host interactions. Comparison of this dataset with previously published datasets helps to define a core secretome present in *Pf*EVs.

**Conclusions**: *P. falciparum *extracellular vesicles contain virulence-associated parasite proteins. Analysis of *Pf*EVs contents from a range of clinical isolates, and their functional validation may improve our understanding of the virulence mechanisms of the parasite, and potentially identify new targets for interventions or diagnostics.

## Introduction

*Plasmodium falciparum* malaria remains a major public health problem, with 212 million cases of malaria and half a million deaths due to severe malaria reported worldwide in 2015 ^1^. The pathogenesis mechanisms of severe malaria are not completely understood, but relevant factors include parasite burden ^2^, induction of host inflammatory responses ^3^, and obstruction of movement of blood in the microvasculature of important organs such as the brain due to adhesion of parasite infected erythrocytes (IEs) to vascular endothelial cells ^4^. Binding of IEs to vascular endothelial cells through endothelial protein C receptor (EPCR) has been hypothesized to cause endothelial activation and inflammation, contributing to pathogenesis ^5, 6^. However, results from other studies suggest that endothelial activation and inflammation during malaria parasite infections can be independent of cytoadhesion of IEs ^7– 9^, and effectors secreted by the parasite may play an important role ^7, 10– 13^.

One way secreted effectors are released from cells is through extracellular vesicles (EVs) that can be classified into two major types; exosomes and microvesicles ^14, 15^. Exosomes are vesicles of endocytic origin with diameter of 30–150 nm. They are generated through inward invagination of the limiting membrane of late endosomes leading to formation of intraluminal vesicles (ILVs) ^16^. During the process of inward invagination of the endosomal membrane, many cytosolic proteins, RNA, and lipids are sorted into the ILVs ^16^. Late endosomes containing multiple ILVs are called multivesicular bodies (MVBs) which when they merge with plasma membrane of the cell, release the ILVs as exosomes into the extracellular space ^17^. By contrast, microvesicles are larger, with a diameter of 100–1000 nm, and are generated through outward invagination of the plasma membrane ^18^. EVs can transfer biologically active effector molecules such as lipids, nucleic acids, metabolites and proteins from one cell to another thereby modifying the properties of the recipient cells ^14, 15, 19, 20^. The physiological significance of these vesicles is becoming increasingly appreciated in many disease processes, including cancer ^15, 21– 23^ and infectious diseases ^12, 24– 28^.

Increasing evidence suggests that EVs play an important role in intercellular communications ^12, 14, 21, 24, 28– 33^. In the context of cancer and infectious diseases, EVs can be used to sabotage the host defence mechanism ^28, 34– 38^, prepare the host cell for invasion ^21, 22, 29, 39, 40^ and acquire nutrients from the environment ^41– 44^. Identifying the content of EVs released by pathogenic organisms will help to understand their basic biology, and potentially identify targets for intervention or diagnostics.

In malaria, *P. falciparum* EVs ( *Pf*EVs) were recently identified in two studies that used long-term adapted laboratory isolates, and proposed that *Pf*EVs play a role in cell-cell communications and gametocytogenesis ^12, 24^. As EVs represent an extended phenotype of the cell ^15^, unravelling the bioactive molecules in *Pf*EVs will contribute to our understanding of the biology and virulence mechanisms of *P. falciparum.* Analogy with other pathogens would suggest that *Pf*EVs may play a role in immunomodulation, nutrient acquisition and invasion *in vivo*. Given that long-term *P. falciparum* laboratory strains, such as 3D7, have not been exposed to the human host environment for decades, it is possible that they may have adapted to release fewer *Pf*EVs or pack a less extensive set of effectors into them, whereas more recently culture adapted *P. falciparum* clinical isolates might release *Pf*EVs containing a greater variety and depth of effectors. Here we present analysis of the protein content of *Pf*EVs isolated from a Kenyan clinical isolate that has been grown *in vitro* for a shorter period than established laboratory strains, and compare it with published *P. falciparum* EV proteome datasets to define a core *Pf*EV content.

## Materials and methods

### Parasite culture

The Kenyan isolate (referred to as isolate 9605) ^45^ was obtained from a child admitted to Kilifi County Hospital with cerebral malaria in 2009. The isolate was adapted to *in vitro* culture and at the time of conducting this study it had been grown *in vitro* for a maximum of 70 cycles, as opposed to many years that most laboratory isolates such as 3D7 have been cultured for. The genome of this isolate was sequenced using Illumina at the Wellcome Trust Sanger Institute, Hinxton, Cambridge and the full genome assembled, (manuscript in preparation). With the publication of the genome, the isolate will be made available to the community through the European malaria reagents repository ( http://www.malariaresearch.eu/reagents), University of Edinburgh. Parasite culture was carried out under standard conditions ^46^.

### Preparation of *P. falciparum* culture-conditioned media

**Figure 1.**
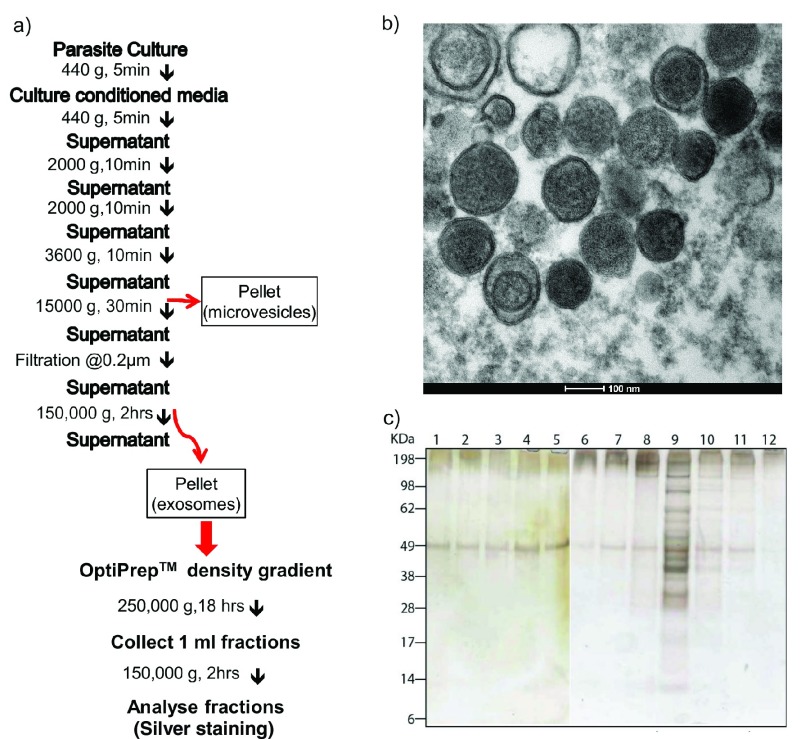
Isolation of *Pf*EVs. **A**). Schematic showing the steps followed to purify *Pf*EVs. **B**). Transmission electron microscopy was used to confirm the presence of vesicles in the pellet. The sizes ranged between 27–411nm (median= 132 nm and mean±SD = 143nm≥66). 73% were below 150nm and 90% were below 200 nm. **C**). Protein extract from each of the density gradient fraction was run on SDS-PAGE gel and stained with silver. The common band is albumin and lanes 9, 10 and 11 corresponding to fractions 10,11 and 12 seem to contain *Pf*EV proteins. This gel only shows analysis of *Pf*EVs from TR time point. The original uncropped image for both RT and TR time points is available at https://osf.io/wdg96/ ^81^.

*P. falciparum* 9605 culture was tightly synchronised by repeated sorbitol treatment and expanded to 6 flasks, each containing 500 µl packed cells at 5–10% parasitemia. RPMI culture media supplemented with AlbumaxII (Gibco) was used to grow the parasite. Albumaxll was depleted of exosomes by centrifuging at 150,000g for 2 hours before addition to *P. falciparum* culture. 50ml of the media was added to each flask when parasites were at early ring stages and harvested after 24 hours when the parasite grew to mature trophozoites (referred to as the ring-to-trophozoite, or RT sample). The culture was then diluted with fresh blood (supplied by NHS, Cambridgeshire, UK) to maintain parasitemia within 5–10% in the following cycle. Fresh media was added and then harvested again 24 hours later, when the parasites had returned to early ring stage (referred to as the trophozoite-to-ring, or TR sample). Culture-conditioned media harvested at each step was processed as outlined in Figure 1. The culture was transferred to 50ml Falcon tubes and centrifuged at 440g for 5 minutes to pellet the erythrocytes. The supernatant culture-conditioned media was centrifuged one more time at 440g for 5 minutes, then twice at 2000g for 10 minutes, once at 3600g for 10 minutes, and finally at 15000g for 30 minutes, each time using new 50ml Falcon tube. The pellet obtained after the 15000g spin was stored at -80°C for future analysis, as it potentially contains microvesicles. The supernatant was filtered at 0.2μm and the flow-through frozen at -80°C until use.

### Purification of *Pf*EVs by density gradient ultracentrifugation

To purify *Pf*EVs, the frozen supernatant generated above was thawed on ice and loaded on quick-seal ultracentrifugation tubes (Bechman Coulter cat# 343322), then centrifuged at 150, 000g for 2hrs using an Optima XE90 ultracentrifuge and 70.1Ti rotor (Beckman Coulter). The pellet was washed twice by re-suspending in cold PBS and centrifuging at 150,000g for 2 hours after each wash. The final pellet was loaded onto OptiPrep™ density gradient medium prepared as described ^47– 49^ and centrifuged at 250,000g for 18 hours. 1ml fractions were collected from the top of the gradient into 1.5ml Eppendorf tubes. To estimate the density of the purified vesicles, the weights of the tubes containing the fractions were measured using weighing machine (Sartorius, PB221S). Each fraction was diluted in PBS to 13.5ml and centrifuged at 150, 000g for 2 hours and the pellet re-suspended in 400µl of 8M urea, 2.5% SDS in 50 mM phosphate buffer, pH 8.0 to extract proteins, before concentrating using a 3KDa MWCO concentrator (Pierce™). A fifth of each fraction was analysed for the presence of proteins by running on SDS-PAGE and staining using silver stain (Bio-Rad).

### Proteomic analysis by mass spectrometry

Fractions confirmed to contain *Pf*EV proteins by silver staining were first reduced by adding DTT to final concentration of 5mM for 10 minutes at 70 **°**C, then alkylated by adding iodoacetamide (IAA) to final concentration of 10 mM and incubated for 30 minutes at room temperature in the dark. The samples were then separated by SDS-PAGE (NuPAGE 4–12% Bis-Tris Gel, Life Technology). The gel was fixed in 40% methanol/2% acetic acid for 30 minutes, stained with colloidal Coomassie (Sigma) overnight, and finally destained with 30% methanol until the background was cleared. Each lane was excised to four pieces, and the gel pieces were destained in 50 mM ammonium bicarbonate/50% CH _3_CN until the gel pieces were completely destained, then they were digested by trypsin (Pierce MS Grade, Thermo Fisher Scientific) overnight at 37 **°**C. Peptides were extracted with 0.5% formic acid (FA)/50% CH _3_CN and dried in a SpeedVac (Thermo Fisher Scientific). The peptides were resuspended in 20μl of 0.5% FA just before LC-MS/MS analysis on a LTQ Orbitrap Velos (Thermo Fisher) hybrid mass spectrometer equipped with a nanospray source, coupled with an Ultimate 3000 RSLCnano System. Samples were first loaded and desalted on a PepMap C18 trap (0.1 mm id × 20 mm, 5µm) at 10µL/min for 15 min, then peptides were separated on a 75 µm id × 25 cm PepMap column (3 µm) at a flow rate of 300 nl/min over a 90 min linear gradient of 4–32% CH _3_CN/0.1% FA, 130 min/cycle. All instrument and columns were from Thermo Fisher Scientific. The LTQ Orbitrap Velos was operated in the “Top 15” data-dependant acquisition mode. The 15 most abundant and multiply-charged precursor ions in the MS survey scan in the Orbitrap (m/z 380 – 1600, with the lock mass at 445.120025) were dynamically selected for CID fragmentation (MS/MS) in the LTQ Velos ion trap. The ions must have a minimal signal above 3000 counts. The preview mode of FT master scan was disabled. The Orbitrap resolution was set at 30,000 at m/z 400 with one microscans. The isolation width for the precursor ion was set at 2 Th. The normalized collision energy was set at 35% with activation Q at 0.250 and activation time for 10 msec. The dynamic exclusion mass width was set at ±10 ppm and exclusion duration for 60 seconds. To achieve high mass accuracy, the AGC (Automatic Gain Control) were set at 1×10 ^6^ for the full MS survey in the Orbitrap with a maximum injection time at 150 msec, and 5000 for the MS/MS in the LTQ Velos with a maximum injection time at 100 msec.

The raw files were processed in MaxQuant (Version 1.5.3.30, www.MaxQuant.org), and searched against both the *Plasmodium falciparum* 3D7 and 9605 protein databases, human protein database (from UniprotKB, October 2014, www.uniprot.org), and a contaminate database supplied by MaxQuant. The mass spectrometry proteomics data have been deposited to the ProteomeXchange Consortium via the PRIDE ^50^ partner repository, with the dataset identifier PXD006925.

Parameters used were mainly in default values, with some modifications: trypsin with maximum 2 missed cleavages sites; peptide mass tolerance at first search was set at 20 ppm and main search was at 4.5 ppm; MS/MS fragment mass tolerance at 0.50 Da, and top 8 MS/MS peaks per 100 Da and a minimum peptide length of 7 amino acids were required. Fixed modification for Carbamidomethyl and variable modification for Acetyl (Protein N-term), Deamidated (NQ) and Oxidation (M) were used. False discovery rates (FDR) were estimated based on matches to reversed sequences in the concatenated target-decoy database. The maximum FDR at 1% was allowed for both proteins and PSMs. Peptides were assigned to protein groups, a cluster of a leading protein(s) plus additional proteins matching to a subset of the same peptides.

### Gene ontology (GO) enrichment analysis

To assess whether certain classes of proteins are enriched in *Pf*EV proteome, gene ontology analysis for enrichment of cellular components was carried out using PlasmoDB ( http://plasmodb.org/plasmo/showApplication.do).

## Results

### Extracellular vesicles were purified from culture conditioned media of a recently adapted *P. falciparum* isolate

We isolated *Pf*EVs from culture-conditioned media of a Kenyan *P. falciparum* clinical isolate adapted to *in vitro* culture. Culture-conditioned media was harvested as shown in Figure 1a, and vesicles pelleted using ultracentrifugation. Examining the pellet by transmission electron microscopy revealed vesicles ( Figure 1b) with median size of 132nm and mean±SD of 143nm±66. This primarily overlaps with the size range of exosomes (30–150nm), but also overlaps with the size range of microvesicles (100–1000nm) and it is likely that both are present. To further purify the pelleted vesicles, the pellet was re-suspended in PBS and subjected to OptiPrep density gradient ultracentrifugation as described in the Methods. 1ml fractions drawn from the top of the gradient were collected, and as shown in Figure 1c, lane 9, 10, and 11 (corresponding to fractions 10, 11, and 12) contained several protein bands, whereas the rest of the fractions contained a single band representing albumin, a common contaminant co-pelleted with EVs. The density of the fractions 10, 11, and 12 ranged between 1.06–1.17g/cm ^3^, consistent with the range described for exosomes (1.08–1.22 g/cm ^3^) ^51^.

### Purified extracellular vesicles contained both host and parasite proteins

To identify the protein content in the isolated *Pf*EVs, fractions identified as containing proteins through silver-staining ( Figure 1c) were run on an SDS-PAGE gel and stained by colloidal Coomassie. Each lane was cut into four pieces and processed for mass spectrometry analysis as described in the Methods. 1194 protein groups were initially identified. Of these, 50 protein groups were potential contaminants (keratin, trypsin, etc), 25 were “Reverse Database” entry (False hits) and 557 were “Only identified by site”. After exclusion of all these groups, 594 protein groups were left for analysis. Of the remaining 594 proteins, the majority of non- *P. falciparum* proteins were of serum/albumax origin such as complement proteins. Only few erythrocyte proteins such as haemoglobin (alpha and beta chains), band 3 anion transport protein, erythrocyte band 7 integral membrane protein, spectrin, glycophorin A and C were identified. 153 *P. falciparum* proteins were also identified ( Table S1) which will be the subject of the subsequent analysis.

### Virulence associated parasite proteins were significantly enriched in the *Pf*EV proteome

We identified 61 and 149 *P. falciparum* proteins in *Pf*EVs purified from the culture conditioned media of ring-to-trophozoite (RT) and trophozoite-to-rings (TR) time points, respectively ( Table S1). 57 of the 61 parasite proteins in the RT time point were also present in the TR, showing reproducibility of enrichment methods and giving a total of 153 *P. falciparum* proteins ( Table S1). The observed high overlap between the RT and TR *Pf*EV proteome may be partly due to the temporal overlap between the two samples at the ring and trophozoite stages, which include the most metabolically active stage where most protein export occurs.

**Figure 2.**
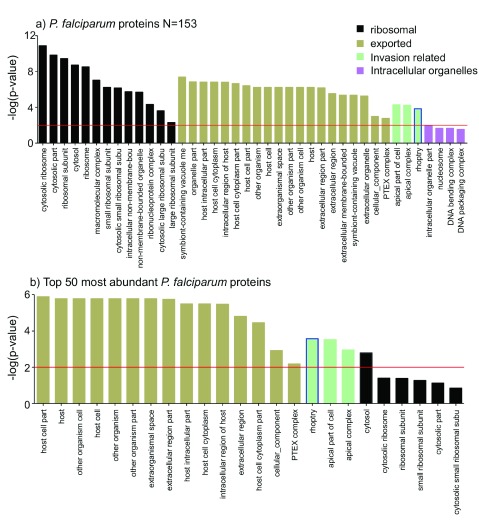
Virulence-associated parasite proteins are significantly enriched in *Pf*EV proteome. **A**). Gene Ontology enrichment analysis for cellular components (N=153). Plotted is the –log p-value (Bonferroni adjusted for 56 comparisons) against the GO terms. The horizontal red line indicates the cut-off for significance (p<0.01). The most significant GO terms were associated with ribosomal, exported and invasion proteins (apical complex). Note the rhoptry proteins (green bar, highlighted with blue line), especially those of the rhoptry bulb were significantly enriched. The rhoptry has been hypothesized to be the equivalent of the endosomal multivesicular body containing vesicles to be secreted to the extracellular environment as exosomes. Enrichment for genes related to intracellular organelles (purple bars) such as mitochondria, ER and nuclear was not significant. **B**). GO-terms associated with the top 50 most abundant proteins (#unique peptide≥4). Plotted is the –log p-value (Bonferroni adjusted for 38 comparisons) against the GO terms. Genes exported to the vesicular compartments within the cytosol of the parasitized red blood cells and those secreted from the endomembrane compartments of the merozoites such as the rhoptry and dense granules were significantly enriched.

GO term enrichment analysis for cellular components revealed that terms related to proteins commonly found in EVs from other systems such as ribosomes ^52, 53^ were also significantly enriched in *Pf*EVs ( Figure 2a). In addition, terms related to virulence associated parasite specific proteins were significantly enriched ( Figure 2a). Here we use the term “virulence associated proteins” to refer to 1) proteins involved in remodelling of the IE such as those exported to cytosol/surface of the IE and 2) proteins involved in invasion of the erythrocytes. These included proteins residents in membrane bound organelles that form in the IE cytosol such as Maurer’s clefts (MCs), and merozoite secretory organelles such as the rhoptry, the microneme and the dense granules ( Figure 2a). By contrast, GO-terms associated with proteins of intracellular organelles such as nucleus, ER, and mitochondria were not significantly enriched in the *Pf*EV proteome ( Figure 2a). Among the apically associated proteins, rhoptry associated proteins were particularly enriched ( Figure 2a). *Pf*EVs from the RT sample were enriched for proteins found in exomembrane compartments of the IEs ( Table S1), but proteins associated with invasion were absent, as expected given the early time point of this sample.

### Parasite proteins exported to the infected erythrocytes and those secreted by the merozoite were the most abundant *Pf*EV proteins

When the list of *Pf*EV proteins was stratified by the number of unique peptides, the top 50 most abundant (≥4 unique peptides) were enriched for parasite proteins that are exported to exomembrane compartments within the infected erythrocytes beyond the parasite plasma membrane ( Figure 2b and Table S1). Proteins found across this exomembrane network, including MCs (SBP1, REX1/2, MAHRP1/2 and MC-2TM), J-dots (HSP40 and HSP70-x), IE membrane (Glag3.1, RhopH3, RESA, KAHRP, *Pf*EMP3) and parasitophorous membrane (PTEX complex, ETRAMP family) were identified ( Table S1). Interestingly while REX1/2, two proteins localised in the MCs ^54^ were detected in the *Pf*EV proteome, REX3, which localises in the IE cytosol ^54^ as a soluble protein, was not detected. Further, *Pf*HRP2 which is secreted into the extracellular space as a soluble protein was also not detected, suggesting most parasite proteins identified in the proteomic analysis are associated with membranes.

Several multigene family proteins such as *Pf*EMP1, Rifins, Stevor, PHIST and FIKK are exported to or via these exomembrane compartments. Among these multigene protein families, PHIST and Rifin families were represented in *Pf*EVs ( Table S1 and Table S2) but no *Pf*EMP1 and Stevor proteins were detected, consistent with previous finding by Mantel *et al.* ^12^. As noted in the Methods, peptide data was searched against both the 3D7 and 9605 proteomes, so the absence of PfEMP1 peptides is likely not due to sequence variation in this highly polymorphic antigen. It should be noted that other proteins that localise at the knobs beneath the IE surface membrane, such as KAHRP and *Pf*EMP3, and proteins linked to transport of *Pf*EMP1 to the surface of the IE (PTP1, PTP6) were found ^55^. These observations suggest that *Pf*EVs are selectively loaded, and do not simply contain a cross section of all exported proteins.

The second most abundant class of parasite proteins enriched in *Pf*EVs was merozoite antigens discharged from the secretory endomembranous compartments of the apical end ( Figure 2b and Table S1). Rhoptry proteins were the most enriched ( Figure 2 and Table S1). These include the RhopH complex (RhopH1 (Clag3.1) RhopH2 and RhopH3), the RAP complex (RAP2 and RAP3), RALP1 and RON3. Notably, these proteins are found in the bulb region of the rhoptry organelle. Except for RON2, proteins from the rhoptry neck region, such as other RONs or members of the reticulocyte binding protein homologue ( *Pf*Rh) family except Rh4, were not detected. Some microneme resident proteins such as EBA-175 and EBA-181 were also present ( Table S1) but notably absent was AMA1, consistent with a previous report ^12^. Several dense granule proteins released during merozoite invasion were also among the most abundant *Pf*EV proteins. These included those that contribute to establishment of a translocon at the PVM for protein export (PTEX members; HSP101, PTEX150, and EXP2) and proteins exported into the invaded erythrocyte early after invasion (SBP1, RESA, MAHRP1).

### Comparing *Pf*EVs datasets to define a core *Pf*EV proteome

**Figure 3.**
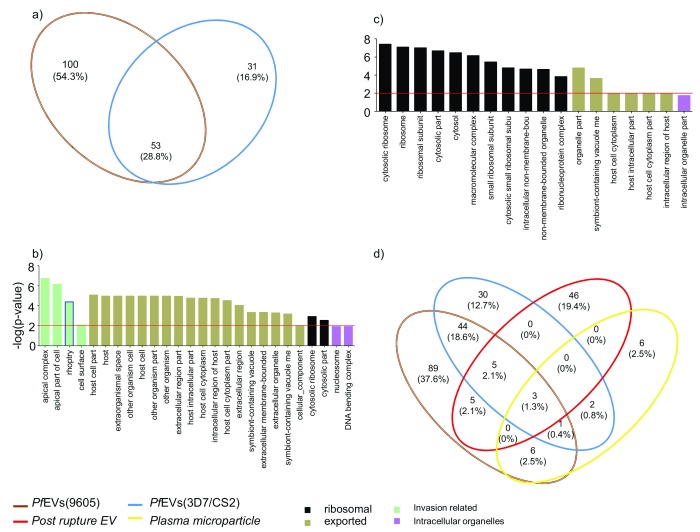
Exported and invasion related *P. falciparum* proteins form the core proteome of *Pf*EVs. **A**) Venn diagram showing that the proteome of *Pf*EVs from the Kenya isolate had extensive overlap with a previously published *Pf*EV proteome (Mantel *et al.* ^12^). **B**) *Pf*EV core proteins: GO-terms enrichment analysis for cellular components of the *Pf*EV proteins common in both the Kenyan and the long-term laboratory isolates used in Mantel *et al.* ^12^. Plotted is the –log p-value (Bonferroni adjusted for 53 comparisons) against the GO terms. **C**) 9605 specific *Pf*EVs proteins: GO-term enrichment analysis showing that the *Pf*EV proteins specific to the Kenyan isolate. Plotted is the –log p-value (Bonferroni adjusted for 46 comparisons) against the GO terms. **D**) Venn diagram showing the overlap between the *Pf*EV proteome identified in 1) this study (dark orange), 2) the previous *Pf*EV proteome (Mantel *et al.* Cell Host Microbe 2013) (blue), 3) Schizont post rupture vesicles (Millholland *et al.* MCP 2011) (red), 4) plasma microparticles from patients with acute *P. falciparum* infection (Antwi-Baffour *et al.* proteome Sci 2017) (yellow).

The proteomic data of the Kenyan clinical isolate had substantial overlap with a previously published *Pf*EV proteome ^12^ ( Figure 3a). Between these two studies 184 *Pf*EV proteins have now been identified ( Figure 3a and Table S2). 53/84 proteins detected by Mantel *et al.* ^12^ were also found in our Kenyan isolate, while 100 proteins (54% of the 184) were unique to the Kenyan isolate ( Figure 3a). GO term enrichment analysis based on cellular components revealed that virulence associated proteins, as defined above, and ribosomal proteins were both significantly enriched in the *Pf*EV proteins shared between the two studies, suggesting that these parasite proteins form the core *Pf*EV proteome ( Figure 3b). However, for the *Pf*EV proteins identified only in the Kenyan isolate, ribosomal and virulence associated exomembrane proteins were significantly enriched, but invasion-related proteins were not ( Figure 3c). Variation between isolates therefore seems to primarily occur in the ribosomal and exported proteins, although it is important to emphasize that technical variation between methodologies of the two studies could also contribute to these differences.

We further compared the *Pf*EV proteome of the Kenyan isolate with two other published proteomes from 1) EVs released during rupture *of P. falciparum* schizonts ^56^ and 2) plasma microparticles isolated from individuals with acute *P. falciparum* infection ^57^. For the post rupture vesicles, we downloaded two files annotated to contain data for sample type “ruptured” ^56^. In these two files, we identified 60 *P. falciparum* proteins ( Table S2), of which 13 were also present in our *Pf*EV proteome ( Figure 3d and Table S2). 8 of the 13 were also present in the *Pf*EV proteome described in Mantel *et al.* ^12^ ( Figure 3d and Table S2). The shared proteins between our study and that of Millholland *et al.* ^57^ were not enriched in invasion-related antigens and only one shared protein, MSP1, was an invasion related antigen ( Table S2). The microparticle proteome from *P. falciparum* infected individuals ^57^ included only 18 *P. falciparum* proteins, of which 10 were present in our *Pf*EV data ( Figure 3d and Table S2). The shared proteins largely consisted of proteins commonly identified in EVs such as heat shock proteins, while the rhoptry proteins enriched in the *Pf*EV proteome of the Kenyan isolate were absent ( Table S2). Therefore, while our data could potentially contain both microparticles or *P. falciparum* schizont post rupture vesicles, the majority of proteins identified in this study have not previously been identified in these two sample types.

## Discussion

In this study, we characterised the protein content of EVs released by a Kenyan *P. falciparum* clinical isolate, 9605. The *Pf*EV purification protocol we used included a centrifugation step at 15000g for 30 min followed by a filtration at 0.2µm ( Figure 1a) to exclude vesicles larger than 200 nm. This step likely led to exclusion of most of the vesicles that fall within what is described as microvesicles that originate from the plasma membrane, and may explain why known host erythrocyte surface antigens were not abundant in our proteomic analysis. However, while the median size of the *Pf*EVs falls within that described for exosomes, it does also overlap microvesicle size ranges, so the data could formally contain both vesicle types.

Proteomics identified 153 *P. falciparum* proteins, of which the most abundant were virulence associated proteins, specifically those involved in erythrocyte invasion and host cell remodelling. Previous proteomic analysis of *Pf*EVs from a long-term laboratory isolate identified 84 *P. falciparum* proteins ^12^, of which 53 were identified again in this study, showing a high degree of overlap. The finding of an additional 100 proteins in this study could be due to technical differences in sample acquisition, vesicle purification or mass spectrometry between the two studies. However, it is also well known that long-term laboratory *P. falciparum* isolates can down-regulate genes involved in pathogenesis *in vivo* but are not required for *in vitro* culture, such as genes associated with cytoadherence. For example, the common laboratory isolate 3D7 predominantly expresses only a limited number of *var* genes ^58^ which encode for the variant surface antigen required for sequestration. Further, genes encoding for exported and sexual stage proteins have been shown to be upregulated in short-term culture adapted clinical isolates (48–86 *in vitro* cycles) as compared to long-term laboratory isolates ^59^. Therefore, it is possible that due to the relatively shorter time the Kenyan isolate spent in *in vitro* culture conditions, 9605- *Pf*EVs proteome may represent a closer reflection of that released by the parasite under *in vivo* conditions. Caution is required due to the technical differences noted above, and the fact that while 9605 isolate is undoubtedly closer to a true clinical isolate than 3D7, it has still been cultured for up to 70 cycles. Analysis of further long and short-term laboratory-adapted isolates processed in exactly the same way will be required to formally test this hypothesis.

The biogenesis of *Pf*EVs is not known, but the proteomic analysis described here revealed several parasite proteins with potential role in biogenesis and transport of endosomal vesicles. These include Rab GTPases ( *Pf*Rab7, *Pf*Rab6), and *P. falciparum* pyruvate kinase (PfPK) ( Table S2), which in other systems are required for transport ^60^ as well as release of exosomes ^61^. The apicomplexan Rab11A has been speculated to be involved in the transport of vesicles derived from endosome-like compartments ^62^. *Pf*EVs also contain several cytosolic proteins commonly found in EVs such as LDH, GAPDH, ENO1, and pyruvate kinase (PK) which are thought to be sorted into ILVs during the formation of MVBs ^16^ suggesting similar mechanism may be involved in the biogenesis of *Pf*EVs.

One of the protein groups most significantly enriched in *Pf*EVs were virulence associated proteins exported to the exomembrane compartments of the IEs ^63^ ( Figure 2), suggesting a proportion of the *Pf*EVs may have an origin in these vesicular compartments. Previous immunoprecipitation of an MC resident protein SEMP1 ^64^, which is also among the most abundant *Pf*EV proteins ( Table S1), co-precipitated 14 proteins of which 13 were identified here as being present in *Pf*EVs ( Table S1/ S2), further reinforcing the link between *Pf*EVs and the exomembrane compartments within the IEs ^63^. Functional analysis of the *P. falciparum* genes described here may therefore shed more light on the process of *Pf*EV biogenesis and release.

Another vesicular compartment closely linked to the contents of the *Pf*EVs is the secretory endomembrane compartments of the merozoite apical end, such as the dense granules and the rhoptry. Comparison of our data with vesicles released post rupture of the schizont showed only a small degree of overlap, so the abundance of these protein classes was unlikely due to the presence of post rupture vesicles among our *Pf*EVs. In particular, proteins from the rhoptry, specifically those from the rhoptry bulb region, were among the most dominant identified ( Table S2), while these were absent from the previously published proteome of schizont post rupture vesicles. The rhoptry bulb has a honeycomb appearance by transmission microscopy, which has been postulated to be formed by internal membranes and/or vesicles ^65^, and it has been further hypothesised that the rhoptry might be the equivalent of multivesicular body (MVB) in higher eukaryotes ^66^. It is therefore possible that the content of the merozoite endomembrane compartments particularly those of the rhoptry and the dense granules are secreted as EVs. This possibility is supported by a recent study that showed 50% of RhopH3 is released into the culture media during merozoite invasion as insoluble membrane-associated protein that can only be pelleted down from the culture media by ultracentrifugation ^67^, which could well be *Pf*EVs. Further, antibody targeting PTEX150 released from dense granules ^68^ precipitated 82 parasite proteins ^69^, of which 42 (51%) are present in *Pf*EVs ( Table S2). The possibility that key components required for early establishment of the exomembrane compartments such as PTEX ^70– 72^, RAP ^73^, and RhopH ^67, 74, 75^ complexes are in part secreted in vesicles will be important to confirm using immunoelectron microscopy.

Screens of large panel of *P. falciparum* proteins using plasma from malaria exposed individuals have identified multiple potential vaccine candidates ^76– 78^. The *Pf*EV proteome contains several of these antigens ( Table S2). *Pf*EVs contain not only parasite proteins, but also TLR agonist such as parasite nucleic acids (Abdi *et al.* unpublished data) that might potentiate immune response ^79^. In this context, immunisation of mice with *P. yoelii* EVs elicited immune response that provided protection against lethal infection ^26, 80^ Further study of *Pf*EVs could therefore be of interest for vaccine development.

## Conclusions

In summary, we have purified *Pf*EVs from a relatively short-term adapted Kenyan isolate. The physical characteristics of the *Pf*EVs primarily overlaps what is known for exosomes, but the sample may also contain microvesicles. Proteomic analysis broadened the previous list of *Pf*EV components, and suggests that a proportion of *Pf*EVs are closely linked to exomembrane and endomembrane compartments in the IEs and merozoites. A major limitation of the study is that a single *P. falciparum* isolate was used and the proteomic data was generated from a single experiment. As such while we can use the data presented in this study to generate hypotheses we cannot make strong conclusions. Therefore, an expanded study of *Pf*EV protein, RNA, lipid and metabolite content from a range of isolates, and functional validation of *Pf*EV components, biogenesis mechanisms, and their role in parasite-parasite and parasite-host interaction is clearly required.

## Data availability

The raw mass spectrometry proteomics data have been deposited to the ProteomeXchange Consortium via the PRIDE ^50^ partner repository, with the dataset identifier PXD006925”. Project name: ‘Proteomic analysis of extracellular vesicles from a Plasmodium falciparum Kenyan clinical isolate defines a core parasite secretome’. The original gel image for Figure 1c is available at https://osf.io/wdg96/ ^81^.

## Ethical statement

Ethical approval was obtained from Kenya Medical Research Institute Scientific and Ethical Review Unit (KEMRI/SERU/CGMRC/022/3149), and written informed consent was obtained from the guardian of the child whose parasite sample was used in this study. The study methods were carried out in accordance with the approved guidelines.

## Supplementary material

**Table S1: List of *P. falciparum* proteins identified in *Pf*EVs.**

' *Pf*EVs_9605_parasite_proteins' contains the list of the proteins ordered in terms of abundance. RT stands for Ring to Trophozoite sample while TR stands for Trophozoite to rings sample. 'GOterms_cellcomp_all' contains GO term analysis based on cellular component. The –log p-value was generated from the Bonferroni adjusted p-value. Part of this analysis is shown graphically in Figure 2a. 'Top50_GOterms_cellcomp' is the GO terms enrichment analysis based on cellular components for the top 50 most abundant proteins. Part of this analysis is shown graphically in Figure 2b.

**Table S2: The combined *Pf*EV proteins.**

'Combined list of *Pf*EVs antigens' are the total number of *Pf*EVs antigens identified in both the Kenyan isolate and the previously published study by Mantel *et al.* ^12^ and some of their characteristics. 'GOterms_cellcomp_ *Pf*EVs_core' is GO-terms enrichment analysis for cellular components of the *Pf*EVs proteins common in both the Kenyan and the long-term laboratory isolates used in Mantel *et al.* ^12^. Part of this analysis is shown graphically in Figure 3b. 'GOterms_cellcomp_9605specific' is the GO-term enrichment analysis showing that the *Pf*EV proteins specific to the Kenyan isolate. Part of this analysis is graphically shown in Figure 3c. Postrupture vesicles (Millholland *et al.* MCP 2011) are the *P. falciparum* proteins identified in the supplementary files named rbc_z01_to_z34.csv and rbc_01_to_10.csv annotated to contain data for the sample type “ruptured”, and plasma microparticles represent proteome of plasma microparticles identified in the study by Antwi-Baffour *et al.* proteome Sci 2017.

## References

[1] WHO: World Malaria Report.2016 Reference Source

[2] Dondorp AM Desakorn V Pongtavornpinyo W: Estimation of the total parasite biomass in acute falciparum malaria from plasma PfHRP2. *PLoS Med.* 2005;2(8):e204. 10.1371/journal.pmed.0020204 16104831 PMC1188247

[3] Cunnington AJ Walther M Riley EM: Piecing together the puzzle of severe malaria. *Sci Transl Med.* 2013;5(211):211ps218. 10.1126/scitranslmed.3007432 24225942

[4] Taylor TE Fu WJ Carr RA: Differentiating the pathologies of cerebral malaria by postmortem parasite counts. *Nat Med.* 2004;10(2):143–145. 10.1038/nm986 14745442

[5] Moxon CA Wassmer SC Milner DA Jr: Loss of endothelial protein C receptors links coagulation and inflammation to parasite sequestration in cerebral malaria in African children. *Blood.* 2013;122(5):842–851. 10.1182/blood-2013-03-490219 23741007 PMC3731936

[6] Turner L Lavstsen T Berger SS: Severe malaria is associated with parasite binding to endothelial protein C receptor. *Nature.* 2013;498(7455):502–505. 10.1038/nature12216 23739325 PMC3870021

[7] Tripathi AK Sha W Shulaev V: *Plasmodium falciparum*-infected erythrocytes induce NF-kappaB regulated inflammatory pathways in human cerebral endothelium. *Blood.* 2009;114(19):4243–4252. 10.1182/blood-2009-06-226415 19713460 PMC2925626

[8] Abdi AI Fegan G Muthui M: *Plasmodium falciparum* antigenic variation: relationships between widespread endothelial activation, parasite PfEMP1 expression and severe malaria. *BMC Infect Dis.* 2014;14:170. 10.1186/1471-2334-14-170 24674301 PMC3986854

[9] Hanson J Lee SJ Hossain MA: Microvascular obstruction and endothelial activation are independently associated with the clinical manifestations of severe falciparum malaria in adults: an observational study. *BMC Med.* 2015;13:122. 10.1186/s12916-015-0365-9 26018532 PMC4453275

[10] Couper KN Barnes T Hafalla JC: Parasite-derived plasma microparticles contribute significantly to malaria infection-induced inflammation through potent macrophage stimulation. *PLoS Pathog.* 2010;6(1):e1000744. 10.1371/journal.ppat.1000744 20126448 PMC2813278

[11] Sun T Holowka T Song Y: A *Plasmodium*-encoded cytokine suppresses T-cell immunity during malaria. *Proc Natl Acad Sci U S A.* 2012;109(31):E2117–2126. 10.1073/pnas.1206573109 22778413 PMC3411961

[12] Mantel PY Hoang AN Goldowitz I: Malaria-infected erythrocyte-derived microvesicles mediate cellular communication within the parasite population and with the host immune system. *Cell Host Microbe.* 2013;13(5):521–534. 10.1016/j.chom.2013.04.009 23684304 PMC3687518

[13] Mantel PY Hjelmqvist D Walch M: Infected erythrocyte-derived extracellular vesicles alter vascular function via regulatory Ago2-miRNA complexes in malaria. *Nat Commun.* 2016;7: 12727. 10.1038/ncomms12727 27721445 PMC5062468

[14] Thery C Ostrowski M Segura E: Membrane vesicles as conveyors of immune responses. *Nat Rev Immunol.* 2009;9(8):581–593. 10.1038/nri2567 19498381

[15] Kalluri R: The biology and function of exosomes in cancer. *J Clin Invest.* 2016;126(4):1208–1215. 10.1172/JCI81135 27035812 PMC4811149

[16] Abels ER Breakefield XO: Introduction to Extracellular Vesicles: Biogenesis, RNA Cargo Selection, Content, Release, and Uptake. *Cell Mol Neurobiol.* 2016;36(3):301–312. 10.1007/s10571-016-0366-z 27053351 PMC5546313

[17] Thery C Zitvogel L Amigorena S: Exosomes: composition, biogenesis and function. *Nat Rev Immunol.* 2002;2(8):569–579. 10.1038/nri855 12154376

[18] Raposo G Stoorvogel W: Extracellular vesicles: exosomes, microvesicles, and friends. *J Cell Biol.* 2013;200(4):373–383. 10.1083/jcb.201211138 23420871 PMC3575529

[19] Schorey JS Cheng Y Singh PP: Exosomes and other extracellular vesicles in host-pathogen interactions. *EMBO Rep.* 2015;16(1):24–43. 10.15252/embr.201439363 25488940 PMC4304727

[20] Zhao H Yang L Baddour J: Tumor microenvironment derived exosomes pleiotropically modulate cancer cell metabolism. *eLife.* 2016;5:e10250. 10.7554/eLife.10250 26920219 PMC4841778

[21] Costa-Silva B Aiello NM Ocean AJ: Pancreatic cancer exosomes initiate pre-metastatic niche formation in the liver. *Nat Cell Biol.* 2015;17(6):816–826. 10.1038/ncb3169 25985394 PMC5769922

[22] Hoshino A Costa-Silva B Shen TL: Tumour exosome integrins determine organotropic metastasis. *Nature.* 2015;527(7578):329–335. 10.1038/nature15756 26524530 PMC4788391

[23] Melo SA Luecke LB Kahlert C: Glypican-1 identifies cancer exosomes and detects early pancreatic cancer. *Nature.* 2015;523(7559):177–182. 10.1038/nature14581 26106858 PMC4825698

[24] Regev-Rudzki N Wilson DW Carvalho TG: Cell-cell communication between malaria-infected red blood cells via exosome-like vesicles. *Cell.* 2013;153(5):1120–1133. 10.1016/j.cell.2013.04.029 23683579

[25] Marti M Johnson PJ: Emerging roles for extracellular vesicles in parasitic infections. *Curr Opin Microbiol.* 2016;32:66–70. 10.1016/j.mib.2016.04.008 27208506 PMC6445373

[26] Martín-Jaular L de Menezes-Neto A Monguió-Tortajada M: Spleen-Dependent Immune Protection Elicited by CpG Adjuvanted Reticulocyte-Derived Exosomes from Malaria Infection Is Associated with Changes in T cell Subsets' Distribution. *Front Cell Dev Biol.* 2016;4:131. 10.3389/fcell.2016.00131 27900319 PMC5110551

[27] Schorey JS Harding CV: Extracellular vesicles and infectious diseases: new complexity to an old story. *J Clin Invest.* 2016;126(4):1181–1189. 10.1172/JCI81132 27035809 PMC4811125

[28] Szempruch AJ Sykes SE Kieft R: Extracellular Vesicles from *Trypanosoma brucei* Mediate Virulence Factor Transfer and Cause Host Anemia. *Cell.* 2016;164(1–2):246–257. 10.1016/j.cell.2015.11.051 26771494 PMC4715261

[29] Peinado H Alečković M Lavotshkin S: Melanoma exosomes educate bone marrow progenitor cells toward a pro-metastatic phenotype through MET. *Nat Med.* 2012;18(6):883–891. 10.1038/nm.2753 22635005 PMC3645291

[30] Thomou T Mori MA Dreyfuss JM: Adipose-derived circulating miRNAs regulate gene expression in other tissues. *Nature.* 2017;542(7642):450–455. 10.1038/nature21365 28199304 PMC5330251

[31] Rayner KJ Hennessy EJ: Extracellular communication via microRNA: lipid particles have a new message. *J Lipid Res.* 2013;54(5):1174–1181. 10.1194/jlr.R034991 23505318 PMC3622315

[32] Li J Liu K Liu Y: Exosomes mediate the cell-to-cell transmission of IFN-α-induced antiviral activity. *Nat Immunol.* 2013;14(8):793–803. 10.1038/ni.2647 23832071

[33] Twu O de Miguel N Lustig G: *Trichomonas vaginalis* exosomes deliver cargo to host cells and mediate host:parasite interactions. *PLoS Pathog.* 2013;9(7):e1003482. 10.1371/journal.ppat.1003482 23853596 PMC3708881

[34] Lambertz U Silverman JM Nandan D: Secreted virulence factors and immune evasion in visceral leishmaniasis. *J Leukoc Biol.* 2012;91(6):887–899. 10.1189/jlb.0611326 22442494

[35] Filipazzi P Bürdek M Villa A: Recent advances on the role of tumor exosomes in immunosuppression and disease progression. *Semin Cancer Biol.* 2012;22(4):342–349. 10.1016/j.semcancer.2012.02.005 22369922

[36] Cestari I Ansa-Addo E Deolindo P: *Trypanosoma cruzi* immune evasion mediated by host cell-derived microvesicles. *J Immunol.* 2012;188(4):1942–1952. 10.4049/jimmunol.1102053 22262654

[37] Aung T Chapuy B Vogel D: Exosomal evasion of humoral immunotherapy in aggressive B-cell lymphoma modulated by ATP-binding cassette transporter A3. *Proc Natl Acad Sci U S A.* 2011;108(37):15336–15341. 10.1073/pnas.1102855108 21873242 PMC3174603

[38] Buck AH Coakley G Simbari F: Exosomes secreted by nematode parasites transfer small RNAs to mammalian cells and modulate innate immunity. *Nat Commun.* 2014;5: 5488. 10.1038/ncomms6488 25421927 PMC4263141

[39] Silverman JM Reiner NE: *Leishmania* exosomes deliver preemptive strikes to create an environment permissive for early infection. *Front Cell Infect Microbiol.* 2012;1:26. 10.3389/fcimb.2011.00026 22919591 PMC3417360

[40] Ghosh J Bose M Roy S: Leishmania donovani targets Dicer1 to downregulate miR-122, lower serum cholesterol, and facilitate murine liver infection. *Cell Host Microbe.* 2013;13(3):277–288. 10.1016/j.chom.2013.02.005 23498953 PMC3605572

[41] Prados-Rosales R Weinrick BC Piqué DG: Role for *Mycobacterium tuberculosis* membrane vesicles in iron acquisition. *J Bacteriol.* 2014;196(6):1250–1256. 10.1128/JB.01090-13 24415729 PMC3957709

[42] Lin J Zhang W Cheng J: A *Pseudomonas* T6SS effector recruits PQS-containing outer membrane vesicles for iron acquisition. *Nat Commun.* 2017;8: 14888. 10.1038/ncomms14888 28348410 PMC5379069

[43] Zhao H Yang L Baddour J: Tumor microenvironment derived exosomes pleiotropically modulate cancer cell metabolism. *eLife.* 2016;5:e10250. 10.7554/eLife.10250 26920219 PMC4841778

[44] Malhotra H Sheokand N Kumar S: Exosomes: Tunable Nano Vehicles for Macromolecular Delivery of Transferrin and Lactoferrin to Specific Intracellular Compartment. *J Biomed Nanotechnol.* 2016;12(5):1101–1114. 10.1166/jbn.2016.2229 27305829

[45] Tan J Pieper K Piccoli L: A *LAIR1* insertion generates broadly reactive antibodies against malaria variant antigens. *Nature.* 2016;529(7584):105–109. 10.1038/nature16450 26700814 PMC4869849

[46] Trager W Jensen JB: Human malaria parasites in continuous culture. *Science.* 1976;193(4254):673–675. 10.1126/science.781840 781840

[47] Tauro BJ Greening DW Mathias RA: Comparison of ultracentrifugation, density gradient separation, and immunoaffinity capture methods for isolating human colon cancer cell line LIM1863-derived exosomes. *Methods.* 2012;56(2):293–304. 10.1016/j.ymeth.2012.01.002 22285593

[48] Van Deun J Mestdagh P Sormunen R: The impact of disparate isolation methods for extracellular vesicles on downstream RNA profiling. *J Extracell Vesicles.* 2014;3(1). 10.3402/jev.v3.24858 25317274 PMC4169610

[49] Xu R Greening DW Rai A: Highly-purified exosomes and shed microvesicles isolated from the human colon cancer cell line LIM1863 by sequential centrifugal ultrafiltration are biochemically and functionally distinct. *Methods.* 2015;87:11–25. 10.1016/j.ymeth.2015.04.008 25890246

[50] Vizcaino JA Csordas A Del-Toro N: 2016 update of the PRIDE database and its related tools. *Nucleic Acids Res.* 2016;44(22):11033. 10.1093/nar/gkw880 27683222 PMC5159556

[51] Raposo G Nijman HW Stoorvogel W: B lymphocytes secrete antigen-presenting vesicles. *J Exp Med.* 1996;183(3):1161–1172. 10.1084/jem.183.3.1161 8642258 PMC2192324

[52] Bosque A Dietz L Gallego-Lleyda A: Comparative proteomics of exosomes secreted by tumoral Jurkat T cells and normal human T cell blasts unravels a potential tumorigenic role for valosin-containing protein. *Oncotarget.* 2016;7(20):29287–29305. 10.18632/oncotarget.8678 27086912 PMC5045396

[53] Dozio V Sanchez JC: Characterisation of extracellular vesicle-subsets derived from brain endothelial cells and analysis of their protein cargo modulation after TNF exposure. *J Extracell Vesicles.* 2017;6(1):1302705. 10.1080/20013078.2017.1302705 28473883 PMC5405560

[54] Spielmann T Hawthorne PL Dixon MW: A cluster of ring stage-specific genes linked to a locus implicated in cytoadherence in *Plasmodium falciparum* codes for PEXEL-negative and PEXEL-positive proteins exported into the host cell. *Mol Biol Cell.* 2006;17(8):3613–3624. 10.1091/mbc.E06-04-0291 16760427 PMC1525250

[55] Maier AG Rug M O´Neill MT: Exported proteins required for virulence and rigidity of *Plasmodium falciparum*-infected human erythrocytes. *Cell.* 2008;134(1):48–61. 10.1016/j.cell.2008.04.051 18614010 PMC2568870

[56] Millholland MG Chandramohanadas R Pizzarro A: The malaria parasite progressively dismantles the host erythrocyte cytoskeleton for efficient egress. *Mol Cell Proteomics.* 2011;10(12):M111 010678. 10.1074/mcp.M111.010678 21903871 PMC3237080

[57] Antwi-Baffour S Adjei JK Agyemang-Yeboah F: Proteomic analysis of microparticles isolated from malaria positive blood samples. *Proteome Sci.* 2017;15:5. 10.1186/s12953-017-0113-5 28352210 PMC5366142

[58] Merrick CJ Dzikowski R Imamura H: The effect of *Plasmodium falciparum* Sir2a histone deacetylase on clonal and longitudinal variation in expression of the *var* family of virulence genes. *Int J Parasitol.* 2010;40(1):35–43. 10.1016/j.ijpara.2009.06.012 19666023

[59] Mackinnon MJ Li J Mok S: Comparative transcriptional and genomic analysis of *Plasmodium falciparum* field isolates. *PLoS Pathog.* 2009;5(10):e1000644. 10.1371/journal.ppat.1000644 19898609 PMC2764095

[60] Savina A Fader CM Damiani MT: Rab11 promotes docking and fusion of multivesicular bodies in a calcium-dependent manner. *Traffic.* 2005;6(2):131–143. 10.1111/j.1600-0854.2004.00257.x 15634213

[61] Wei Y Wang D Jin F: Pyruvate kinase type M2 promotes tumour cell exosome release via phosphorylating synaptosome-associated protein 23. *Nat Commun.* 2017;8: 14041. 10.1038/ncomms14041 28067230 PMC5228053

[62] Agop-Nersesian C Naissant B Ben Rached F: Rab11A-controlled assembly of the inner membrane complex is required for completion of apicomplexan cytokinesis. *PLoS Pathog.* 2009;5(1):e1000270. 10.1371/journal.ppat.1000270 19165333 PMC2622761

[63] Sherling ES van Ooij C: Host cell remodeling by pathogens: the exomembrane system in *Plasmodium*-infected erythrocytes. *FEMS Microbiol Rev.* 2016;40(5):701–721. 10.1093/femsre/fuw016 27587718 PMC5007283

[64] Dietz O Rusch S Brand F: Characterization of the small exported *Plasmodium falciparum* membrane protein SEMP1. *PLoS One.* 2014;9(7):e103272. 10.1371/journal.pone.0103272 25062022 PMC4111544

[65] Bannister LH Mitchell GH Butcher GA: Lamellar membranes associated with rhoptries in erythrocytic merozoites of *Plasmodium knowlesi*: a clue to the mechanism of invasion. *Parasitology.* 1986;92(Pt 2):291–303. 10.1017/S0031182000064064 2423944

[66] Yang M Coppens I Wormsley S: The *Plasmodium falciparum Vps4* homolog mediates multivesicular body formation. *J Cell Sci.* 2004;117(Pt 17):3831–3838. 10.1242/jcs.01237 15252121

[67] Ito D Schureck MA Desai SA: An essential dual-function complex mediates erythrocyte invasion and channel-mediated nutrient uptake in malaria parasites. *eLife.* 2017;6: pii: e23485. 10.7554/eLife.23485 28221136 PMC5349850

[68] Bullen HE Charnaud SC Kalanon M: Biosynthesis, localization, and macromolecular arrangement of the *Plasmodium falciparum* translocon of exported proteins (PTEX). *J Biol Chem.* 2012;287(11):7871–7884. 10.1074/jbc.M111.328591 22253438 PMC3318755

[69] Elsworth B Sanders PR Nebl T: Proteomic analysis reveals novel proteins associated with the *Plasmodium* protein exporter PTEX and a loss of complex stability upon truncation of the core PTEX component, PTEX150. *Cell Microbiol.* 2016;18(11):1551–1569. 10.1111/cmi.12596 27019089

[70] Elsworth B Matthews K Nie CQ: PTEX is an essential nexus for protein export in malaria parasites. *Nature.* 2014;511(7511):587–591. 10.1038/nature13555 25043043

[71] Beck JR Muralidharan V Oksman A: PTEX component HSP101 mediates export of diverse malaria effectors into host erythrocytes. *Nature.* 2014;511(7511):592–595. 10.1038/nature13574 25043010 PMC4130291

[72] de Koning-Ward TF Gilson PR Boddey JA: A newly discovered protein export machine in malaria parasites. *Nature.* 2009;459(7249):945–949. 10.1038/nature08104 19536257 PMC2725363

[73] Ghosh S Kennedy K Sanders P: The *Plasmodium rhoptry* associated protein complex is important for parasitophorous vacuole membrane structure and intraerythrocytic parasite growth. *Cell Microbiol.* 2017;19(8):e12733. 10.1111/cmi.12733 28205319

[74] Counihan NA Chisholm SA Bullen HE: *Plasmodium falciparum* parasites deploy RhopH2 into the host erythrocyte to obtain nutrients, grow and replicate. *eLife.* 2017;6: pii: e23217. 10.7554/eLife.23217 28252383 PMC5365316

[75] Sherling ES Knuepfer E Brzostowski JA: The *Plasmodium falciparum* rhoptry protein RhopH3 plays essential roles in host cell invasion and nutrient uptake. *eLife.* 2017;6: pii: e23239. 10.7554/eLife.23239 28252384 PMC5365315

[76] Crompton PD Kayala MA Traore B: A prospective analysis of the Ab response to *Plasmodium falciparum* before and after a malaria season by protein microarray. *Proc Natl Acad Sci U S A.* 2010;107(15):6958–6963. 10.1073/pnas.1001323107 20351286 PMC2872454

[77] Torres KJ Castrillon CE Moss EL: Genome-level determination of *Plasmodium falciparum* blood-stage targets of malarial clinical immunity in the Peruvian Amazon. *J Infect Dis.* 2015;211(8):1342–1351. 10.1093/infdis/jiu614 25381370 PMC4402338

[78] Dent AE Nakajima R Liang L: *Plasmodium falciparum* Protein Microarray Antibody Profiles Correlate With Protection From Symptomatic Malaria in Kenya. *J Infect Dis.* 2015;212(9):1429–1438. 10.1093/infdis/jiv224 25883384 PMC4601912

[79] Kasturi SP Skountzou I Albrecht RA: Programming the magnitude and persistence of antibody responses with innate immunity. *Nature.* 2011;470(7335):543–547. 10.1038/nature09737 21350488 PMC3057367

[80] Martin-Jaular L Nakayasu ES Ferrer M: Exosomes from *Plasmodium yoelii*-infected reticulocytes protect mice from lethal infections. *PLoS One.* 2011;6(10):e26588. 10.1371/journal.pone.0026588 22046311 PMC3202549

[81] Abdi A: Proteomic analysis of extracellular vesicles from a *Plasmodium falciparum* Kenyan clinical isolate.2017 Data Source 10.12688/wellcomeopenres.11910.2 PMC5583745 28944300

