# Supplementary material for: Proteomic analysis of extracellular vesicles from a Plasmodium falciparum Kenyan clinical isolate defines a core parasite secretome
Source: Wellcome Open Res. 2017 Nov 22;2:50. Originally published 2017 Jul 17. [Version 2] doi: 10.12688/wellcomeopenres.11910.2 (PMC5583745; doi:10.12688/wellcomeopenres.11910.2)
Supplement: Peer review history

# Peer review: Referee response for version 2

**DOI:** 10.21956/wellcomeopenres.14296.r28263

**Published:** 2018-01-05

**Authors:** Pawan Malhotra, Inderjeet Kaur

**Affiliations:** 1 Malaria Biology Group, International Centre for Genetic Engineering and Biotechnology (ICGEB), New Delhi, Delhi , India

We accept the manuscript with reservation as authors have replied positively to most of our queries. However, since proteome analysis has been performed on limited samples, we think that some of the experiment(s) that we suggested are doable as author(s) could have raised some antibodies for published exosome markers and performed the co-localization studies/western studies.

We have read this submission. We believe that we have an appropriate level of expertise to confirm that it is of an acceptable scientific standard, however we have significant reservations, as outlined above.

# Peer review: Referee response for version 2

**DOI:** 10.21956/wellcomeopenres.14296.r28264

**Published:** 2017-12-08

**Authors:** Matthias Marti

**Affiliations:** 1 Wellcome Trust Center for Molecular Parasitology, University of Glasgow, Glasgow, UK

The revised version of this MS has been significantly improved and addressed most concerns.  In particular additional comparison with previously published studies have been added. My only remaining comment is that there is still no clear statement as  to what the different vesicle populations might be.

 Given the sampling regime and their different composition, I would assume that they are a mixture of iRBC-derived microvesicles and materials released during egress invasion ("exosomes"). It would be helpful if this could be clarified in the discussion.

I have read this submission. I believe that I have an appropriate level of expertise to confirm that it is of an acceptable scientific standard.

# Peer review: Referee response for version 1

**DOI:** 10.21956/wellcomeopenres.12873.r24514

**Published:** 2017-09-01

**Authors:** Alister G. Craig, Climent Casals-Pascual

**Affiliations:** 1 Department of Parasitology, Liverpool School of Tropical Medicine, Liverpool, UK; 2 Nuffield Department of Medicine, Wellcome Trust Centre for Human Genetics, University of Oxford, Oxford, UK

This paper extends the work of Mantel *et al* ^1^ using an isolate of *Plasmodium falciparum* more recently derived from patient material.  Microvesicles, or extracellular vesicles (EV) have previously been shown to activate immune cells and trigger gametocytogenesis, and a deeper understanding of the proteins displayed by them could support further hypotheses about their role *in vivo*.  The data presented here increases the list of proteins identified and suggests that EV are derived from internal vesicular structures rather than the infected erythrocyte membrane. 

 Comments:

1. The term “clinically relevant” in the abstract is not supported by any clinical data about the infection the parasite line 9605 was derived from.  Additionally, after 70 cycles of *in vitro* culture, while this isolate is closer to its patient origins than 3D7 it is not known how closely it still represents the original sample.  This is acknowledged by the authors later in the paper and will require further work to define the changes that occur during culturing.  For example, it may be possible that parasites reduce the release of EV or alter the spectrum of proteins released, but no data are provided to support this other than the single 9605 line.
2. What was the rationale to use “fractionated” in-gel digestion for (membrane-rich) EVs? Did the authors try alternative protocols that facilitate the identification of membrane-bound proteins such as FASP (filter-aided sample preparation)?
3. What was the proportion of “contaminants” (false hits) that were excluded from the analysis?
4. The potential role of EV in malaria infections is an exciting field but the data supporting their direct effect on endothelial activation, independent of cytoadherence, are limited.  Of the references cited (#7, 10-12) two are from mouse studies, one does not identify whether EV were involved in the *in vitro* model system used and one shows EV activation of monocytes and neutrophils but not endothelium. 
5. The finding of 57 out of 61 parasite proteins from the RT sample being in the TR sample might show some reproducibility but could also be considered surprising given the temporal regulation of expression seen in the erythrocytic cycle of *P. falciparum*.   Does this suggest that proteins exported to the erythrocytic cytoplasm and vesicles within this compartment are retained for the whole cycle despite relatively stringent control of mRNA production? 
6. Could the lack of discovery of PfEMP1 peptides be related to their absence from the 9605 genome sequence available for proteomic analysis?  How well did the subtelomeric regions assemble for this isolate?  The pool of this protein within the cell is thought to be significant and it has been associated with the Maurer’s Clefts, so its lack is interesting. 
7. Some comment on the dominance of ribosomal proteins in the list of hits specifically identified in this study, compared to Mantel et al, would be useful.  Is this a technical issue?  How might this be interpreted in terms of the origin of EV?
8. The presence of virulence proteins in exosomes appears to be one of the major findings in this manuscript. Perhaps, the authors should detail the criteria used to include proteins under “virulence-associated parasite proteins”.
9. The point being made in the final paragraph of the discussion is not entirely clear.  The display of internal parasite antigens by EV could explain exposure of these to the immune system, but this is also covered by the rupture of infected erythrocytes.  What is the specific role of EV in this context?  Some clarification would be useful.  Along similar lines, what is meant in the conclusions about “fine-tuning the host immune response, preparing the host cell for invasion and acquiring nutrients from the environment”.  Which parts of the dataset contribute to these suggestions? 
10. In the supplementary material, it would be useful to have a column with the common names of proteins in Table S1, as shown in Table S2 under “product description”. 
11. Have any of the proteins identified in the proteomic analysis been validated using an independent method, like Western blot or enzyme immunoassays? 

Perhaps the major limitation of the study is that the proteomic description of EVs is limited to a single isolate and a single “experiment” / culture. It would have been desirable to see a similar analysis for few clinical isolates or, at least, in more than one culture from the same isolate, although this is partially mitigated through the comparison with previously published data.  This would have been helpful to appreciate the experimental and biological variability. It is not uncommon for proteomics (or any – *omics*) studies to generate a number of interesting hits (proteins of interest) followed by hypotheses or plausible explanations about the biological phenomenon under study. The risks associated with this approach (sharpshooter fallacy) can be partially circumvented by proposing hypotheses that can be tested experimentally or by validating the mass spectrometry findings using alternative methods.  What would be the single most important experiment or that would follow logically from the description provided in the manuscript?

We have read this submission. We believe that we have an appropriate level of expertise to confirm that it is of an acceptable scientific standard.

## References

[1] MantelPYHoangANGoldowitzIPotashnikovaDHamzaBVorobjevIGhiranITonerMIrimiaDIvanovARBartenevaNMartiM: Malaria-infected erythrocyte-derived microvesicles mediate cellular communication within the parasite population and with the host immune system. *Cell Host Microbe*.2013;13(5) : 10.1016/j.chom.2013.04.009 521-34 10.1016/j.chom.2013.04.009 23684304PMC3687518

# Peer review: Referee response for version 1

**DOI:** 10.21956/wellcomeopenres.12873.r24571

**Published:** 2017-08-17

**Authors:** Pawan Malhotra, Inderjeet Kaur

**Affiliations:** 1 Malaria Biology Group, International Centre for Genetic Engineering and Biotechnology (ICGEB), New Delhi, Delhi , India

The study by Abdi *et al* illustrates the proteome of extracellular vesicles from the clinical isolates that have not passed through many passages. The study is important and provides additional information than previously published couple of studies describing the exosomes proteomes. I recommend indexing after major modifications. Some specific comments are as follows:

 1. Authors should have used some known exosome markers to show the purity of vesicles. They could have carried-out western blot or immunofluorescence analysis using the specific exosome protein antibodies to prove this point.

 2. Like-wise authors would have used similar techniques to confirm the presence of additional proteins that they found in their analysis.

 3.It is not clear that how many biological replicates were used in the present study.

 4. I also think a comparison between earlier studies and present study could have been represented by Venn diagram.

 5. Authors could have used some markers to prove their hypothesis that EVs are not derived from infected erythrocyte membranes.

We have read this submission. We believe that we have an appropriate level of expertise to confirm that it is of an acceptable scientific standard, however we have significant reservations, as outlined above.

# Peer review: Referee response for version 1

**DOI:** 10.21956/wellcomeopenres.12873.r24252

**Published:** 2017-07-28

**Authors:** Matthias Marti

**Affiliations:** 1 Wellcome Trust Center for Molecular Parasitology, University of Glasgow, Glasgow, UK

The study by Abdi *et al*. describes proteomic analysis of *P. falciparum*-derived extracellular vesicles from a clinical isolate. The presented data expand our knowledge of the protein content of these vesicles and provide a basis for diagnostic and functional studies.

 The manuscript is well written, however the data analysis and presentation is lacking some detail that would be helpful for interpretation. Most importantly the authors conclude they have analyzed exosomes originating from apical organelles rather than EVs derived from the IE surface. This may be true, see also comments below, but then the title and description of data should be altered.

 Some specific comments below:

  

- There is no systematic comparison between RT and TR proteome. It is assumed that the TR-specific proteome is enriched in egress material but that remains to be confirmed (see below).
- The TR sample includes egress and invasion events. Therefore materials released during this phase will be present in the sample. An egress proteome has been published ^1^ and should be included in the comparative analysis.
- Note that recently another EV proteome was published that should also be included in this analysis ^2^.
- In the comparison with the proteome data from Mantel *et al* the majority of additional proteins is ribosomal (Figure 3c). These are unlikely vesicle-derived but rather contaminants. Is there an explanation for this? 
- The conclusion that the Kenyan strain contains a larger variety of EV proteins in comparison to 3D7/CS2 cannot be drawn as different populations of EVs (with this study including egress vesicles) were analysed.
- PHIST and Rifin proteins are detected in EVs but it is concluded EVs are not derived from the IE surface. This would be surprising since the previous analysis also included RBC proteins and detected a number of surface antigens including glycophorins. Was host proteome also investigated here?
- It is mentioned that many merozoite antigens are detected, mostly from rhoptries but also micronemes. This would again suggest egress vesicles  (or what the authors call exosomes) rather than vesicles released during between invasion and egress.
- The authors conclude that they may have analyzed exosomes rather than EVs, and that exosomes may form from apical organelles. The argument that EVs can only be exosomes is not conclusive. The authors argue that EVs cannot be derived from the erythrocyte despite identifying components of the erythrocyte membrane in their proteome (e.g. band 3, top right paragraph on page 5). Selective cargo loading of *P. falciparum* proteins might occur at the erythrocyte membrane and thus explain why only some RBC membrane localized parasite proteins are detected in EVs.
- The authors conclude that EVs may derive from Maurer’s clefts as several Maurer’s clefts proteins were identified. In combination with their conclusion that PfEVs are only comprised of exosomes, do the authors suggest that Maurer’s clefts are multivesicular body like compartments? Are there any reports about vesicles inside Maurer’s clefts? 

I have read this submission. I believe that I have an appropriate level of expertise to confirm that it is of an acceptable scientific standard, however I have significant reservations, as outlined above.

## References

[1] MillhollandMGChandramohanadasRPizzarroAWehrAShiHDarlingCLimCTGreenbaumDC: The malaria parasite progressively dismantles the host erythrocyte cytoskeleton for efficient egress. *Mol Cell Proteomics*.2011;10(12) : 10.1074/mcp.M111.010678 M111.010678 10.1074/mcp.M111.010678 21903871PMC3237080

[2] Antwi-BaffourSAdjeiJKAgyemang-YeboahFAnnani-AkollorMKyeremehRAsareGAGyanB: Proteomic analysis of microparticles isolated from malaria positive blood samples. *Proteome Sci*.2016;15: 10.1186/s12953-017-0113-5 5 10.1186/s12953-017-0113-5 28352210PMC5366142
